# Identification of diagnosis biomarkers based on the delirium-related genes

**DOI:** 10.1097/JS9.0000000000000874

**Published:** 2023-11-07

**Authors:** Xing-Long Xiong, Shi-Jie Tang, Jing Shi

**Affiliations:** Department of Anesthesiology, The Affiliated Hospital of Guizhou Medical University, Guiyang, People’s Republic of China

*Dear Editor*,

Delirium, a profound neuropsychiatric syndrome, emerges acutely, presenting attention deficits and additional cognitive dysfunctions not attributable to prior neurocognitive impairments. Delirium is a significant risk factor for reduced quality of life for patients with acute and critical illnesses due to persistent cognitive dysfunction, functional disability, and deteriorating mental health. According to an article published in the *International Journal of Surgery*, there are several widely examined and consistent risk factors associated with delirium. These include increasing age, residency in nursing homes, pre-existing cognitive impairment, psychiatric disorders, cerebrovascular disease, end-stage renal failure, low albumin levels, poor functional status, and intra-operative blood transfusion^1^. These factors contribute to the occurrence of various neurobiological processes in the pathogenesis of delirium, such as neuroinflammation, cerebrovascular dysfunction, altered brain metabolism, neurotransmitter imbalance, and impaired neuronal network connectivity^2,3^. Despite the available pharmacological therapies, such as antipsychotic medications, they have proven to be ineffective in treating delirium. Therefore, the main objective of this study is to assess the molecular-level pathogenesis of delirium in order to gain a better understanding of its role in both physiology and pathology.

Initially, 135 delirium-related genes (Inference Score >60) were extracted from the Comparative Toxicogenomics Database (CTD) (https://ctdbase.org)^4^. Subsequent Gene Ontology (GO) and Kyoto Encyclopedia of Genes and Genomes (KEGG) pathway enrichment analyses highlighted several key pathways associated with delirium risk, such as inflammation, immune cell migration, apoptosis, angiogenesis, synaptic inhibition, and neuronal cell death, as visualized in Figure 1 (A, B). In individuals at high risk, delirium is a manifestation of the vulnerable brain’s inability to withstand a sudden stressor. This dysfunction can result from multiple processes that are not mutually exclusive. Firstly, the connections in the brain’s network become weakened as a result of aging and neurodegeneration. Secondly, research using animal models has shown that microglia and astrocytes, which are already affected by neurodegeneration, generate a cascading pro-inflammatory response to subsequent inflammatory stimuli, thereby amplifying inflammation. Thirdly, aging and neurodegeneration bring about changes in the cerebrovascular system, which may increase the brain’s susceptibility to disruptions in the supply of energy and oxygen, as well as the circulation of inflammatory molecules^3^. Proteomic data for GSE242736 were obtained from the Gene Expression Omnibus (GEO) database for further validation. Twenty-four matched delirium cases and non-delirium controls were selected from the Healthier Postoperative Recovery (HiPOR) cohort. Data from this cohort measured 1305 proteins in preoperative cerebrospinal fluid and analysis identified 32 proteins in preoperative cerebrospinal fluid significantly associated with delirium (*t*-test *P*<0.05). A subsequent analysis using KEGG and GO enrichment, depicted in Supplementary Figure 1 (A, B) (Supplemental Digital Content 1, http://links.lww.com/JS9/B313), revealed analogous enrichment in inflammation, immune cell migration, apoptosis, and synaptic inhibition among these differential proteins. Intersecting the 135 delirium-related genes with the 32 differential proteins yielded 14 key delirium genes (‘CCL2,’ ‘NGF,’ ‘INS,’ ‘CTSD,’ ‘PRKCA,’ ‘IGFBP2,’ ‘NAMPT,’ ‘PARK7,’ ‘FSTL1,’ ‘MMP14,’ ‘ACAN,’ ‘DCN,’ ‘PTPN6,’ ‘CFL1’), with their pathway relationships illustrated in Figure 1C. Additionally, hierarchical clustering identified these 14 genes, distinguishing between delirium and normal populations in Figure 1D.

**Figure 1:**
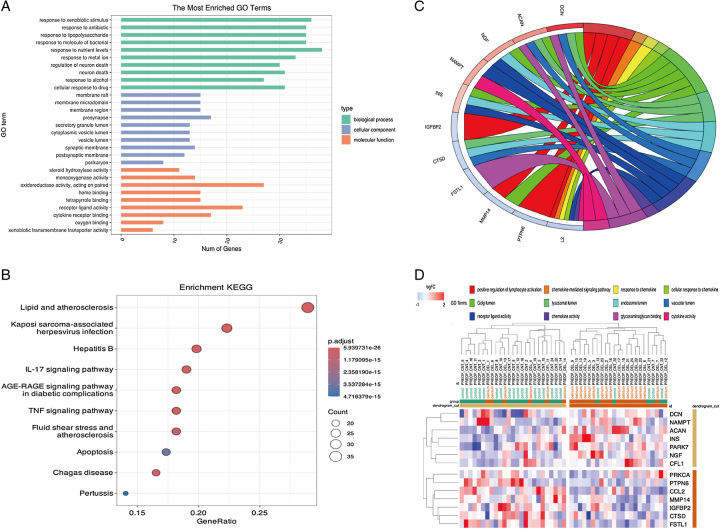
Enrichment analysis. (A) GO enrichment results of 135 genes from CTD. (B) KEGG enrichment results of 135 genes from CTD. (C) GOChord map of 14 key delirium genes (intersection of CTD and GEO differential genes). (D) Hierarchical clustering based on 14 key delirium genes (intersection of CTD and GEO differential genes). CTD, Comparative Toxicogenomics Database; GEO, Gene Expression Omnibus; GO, Gene Ontology; KEGG, Kyoto Encyclopedia of Genes and Genomes.

The lack of success in current pharmacotherapy research may be attributed to the diverse nature of delirium. It is possible that a specific drug intervention that proves effective in one individual with delirium may not yield the same results in another individual due to the fact that the underlying physiological processes leading to delirium can vary significantly. This finding suggests that the 14 identified genes could potentially serve as molecular markers for screening delirium, providing a strong foundation for scientific evaluation and targeted interventions specifically designed for delirium populations. In conclusion, delirium is a prevalent and serious complication following surgery, and unfortunately, there are limited established measures for its prevention^5^.

## Ethical approval

GEO belongs to public databases. The patients involved in the database have obtained ethical approval. Users can download relevant data for free for research and publish relevant articles. Our study is based on open source data, so there are no ethical issues and other conflicts of interest.

## Consent

Our study is based on open source data, so there are no ethical issues and informed written consent.

## Sources of funding

This work was supported by the National Natural Science Foundation of China (Grant No. 82060220 to J.S.) and the Science and Technology Plan Project of Guizhou province [ZK (2023) – General 390 to J.S.].

## Author contribution

S.-J.T. and X.-L.X.: study concept, design, and acquisition of data; X.-L.X. and S.-J.T.: data cleaning and analysis, and interpreted the data; X.-L.X. and S.-J.T.: drafting the manuscript; X.-L.X. and S.-J.T.: contributed equally to this study. All the authors approved the final manuscript as submitted and agreed to be accountable for all aspects of the work.

## Conflicts of interest disclosure

The authors declare no conflicts of interest.

## Research registration unique identifying number (UIN)

Our study is based on open source data, so there is no research registration.

## Guarantor

All authors.

## Data availability statement

The data presented in this study are available on request from the corresponding author.

## Provenance and peer review

Not commissioned, externally peer-reviewed.

## Supplementary Material
