# Supplementary material for: Identification of diagnosis biomarkers based on the delirium-related genes
Source: Int J Surg. 2023 Nov 7;110(2):1291–3. doi: 10.1097/JS9.0000000000000874 (PMC10871556; doi:10.1097/JS9.0000000000000874)
Supplement: Supplementary file 1 [file js9-110-1291-s001.docx]

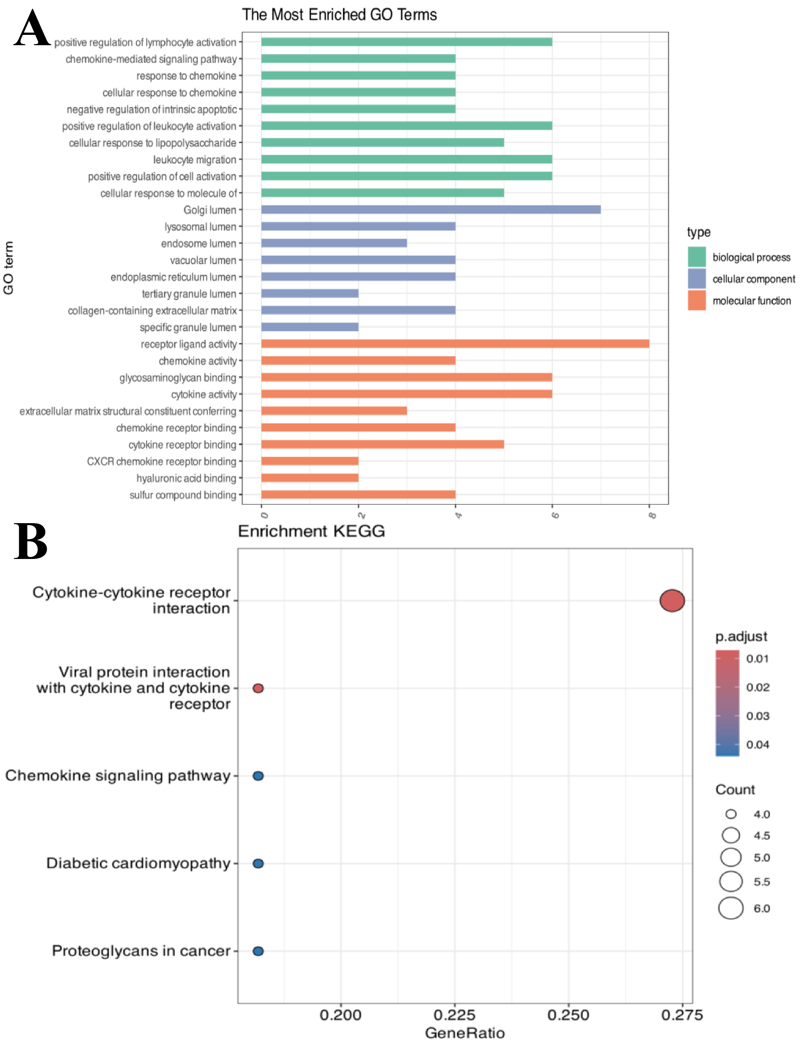
**Supplementary Figure1. Enrichment analysis. (A) GO enrichment results of 32 difference genes from GSE242736. (B) KEGG enrichment results of 32 difference genes from GSE242736.**
